# The Transcription Factor NRF2 Has Epigenetic Regulatory Functions Modulating HDACs, DNMTs, and miRNA Biogenesis

**DOI:** 10.3390/antiox12030641

**Published:** 2023-03-04

**Authors:** Ignacio Silva-Llanes, Chang Hoon Shin, José Jiménez-Villegas, Myriam Gorospe, Isabel Lastres-Becker

**Affiliations:** 1 Instituto de Investigaciones Biomédicas “Alberto Sols” UAM-CSIC, Arturo Duperier, 4, 28029 Madrid, Spain; 2 Instituto de Investigación Sanitaria La Paz (IdiPaz), 28046 Madrid, Spain; 3 Laboratory of Genetics and Genomics, National Institute on Aging Intramural Research Program, National Institutes of Health, Baltimore, MD 21224, USA; 4 Department of Biochemistry, School of Medicine, Universidad Autónoma de Madrid, 28029 Madrid, Spain; 5 Centro de Investigación Biomédica en Red de Enfermedades Neurodegenerativas (CIBERNED), Instituto de Salud Carlos III, 28031 Madrid, Spain; 6 Institute Teófilo Hernando for Drug Discovery, Universidad Autónoma de Madrid, 28029 Madrid, Spain

**Keywords:** NRF2, epigenetics, HDAC, DNMT, miRNA, TDMD, DMF, oxidative stress

## Abstract

The epigenetic regulation of gene expression is a complex and tightly regulated process that defines cellular identity and is associated with health and disease processes. Oxidative stress is capable of inducing epigenetic modifications. The transcription factor NRF2 (nuclear factor erythroid-derived 2-like 2) is a master regulator of cellular homeostasis, regulating genes bearing antioxidant response elements (AREs) in their promoters. Here, we report the identification of ARE sequences in the promoter regions of genes encoding several epigenetic regulatory factors, such as histone deacetylases (HDACs), DNA methyltransferases (DNMTs), and proteins involved in microRNA biogenesis. In this research, we study this possibility by integrating bioinformatic, genetic, pharmacological, and molecular approaches. We found ARE sequences in the promoter regions of genes encoding several HDACs, DNMTs, and proteins involved in miRNA biogenesis. We confirmed that NRF2 regulates the production of these genes by studying NRF2-deficient cells and cells treated with dimethyl fumarate (DMF), an inducer of the NRF2 signaling pathway. In addition, we found that NRF2 could be involved in the target RNA-dependent microRNA degradation (TDMD) of miR-155-5p through its interaction with *Nfe2l2* mRNA. Our data indicate that NRF2 has an epigenetic regulatory function, complementing its traditional function and expanding the regulatory dimensions that should be considered when developing NRF2-centered therapeutic strategies.

## 1. Introduction

The cells of multicellular organisms have the same genetic content, yet their functions are diverse because they express different genes. These differences are due in large part to epigenetic mechanisms that lead to heritable and stable changes in gene expression programs that occur through alterations in chromatin structure. Epigenetic gene regulation provides an adaptive layer of control of gene expression to enable the organism to adjust to a varying environment by eliciting histone modifications, DNA methylation, and gene silencing via microRNAs (miRNAs) [1,2]. Epigenetic mechanisms are not only key in the processes of cell development and differentiation, but they also play important roles in many pathologies, including neurodegenerative diseases.

Oxidative stress arises from an imbalance between reactive oxygen species and the cell’s antioxidant capacity, leading to an accumulation of ROS and a disruption of the epigenetic state of the cell. In turn, oxidative damage triggers epigenetic changes in the chromatin structure, histone modifications, DNA methylation, and DNA-binding proteins [3]. One of the ways for the cell to combat oxidative stress is by activating the NRF2 (nuclear factor erythroid-derived 2-like 2) transcription factor signaling pathway. NRF2 is an essential factor that transcriptionally regulates over 250 genes involved in the antioxidant response, biotransformation reactions, mitochondrial bioenergetics, inflammation, and proteostasis, among others [4,5,6,7]. Because NRF2 is able to regulate so many genes and therefore has a considerable impact on a wide range of cellular functions, its expression tightly regulated. In basal conditions, there are low levels of NRF2 due to the action of an E3 ubiquitin ligase complex containing a substrate adaptor protein, Kelch-like ECH-associated protein 1 (KEAP1), which binds to and negatively regulates NRF2 [8,9]. However, in conditions in which there is a significant increase in oxidative stress, NRF2 signaling is induced through modifications of key cysteine residues in KEAP1, which induce conformational changes in the binding of NRF2–KEAP1 and prevent the degradation of NRF2. This allows for the accumulation of newly synthesized NRF2, which can then translocate to the nucleus, binds the antioxidant response element (ARE) sequence in the promoter regions of NRF2-dependent genes, and recruits the transcriptional machinery [9,10,11]. The rise in NRF2 expression can also be elicited at the epigenetic level; for example, hypermethylation of the first five CpG sites in the NRF2 promoter was associated with mouse prostate tumorigenesis [12] and the frequency of demethylation was significantly higher in colorectal cancer [13]. Further, recent evidence has identified several miRNAs that can be regulated by NRF2 in the context of several pathologies, such as cancer [14], cardiovascular diseases [15], and neurodegenerative diseases [16].

However, until now, whether NRF2 influences epigenetic processes by modulating the expression of DNA methyltransferases (DNMTs), histones deacetylases (HDACs), and miRNAs has not been investigated. Here, we identify for the first time several HDACs, DNMTs, and genes encoding proteins involved in the biogenesis of miRNAs. Additionally, we present evidence that NRF2 could modulate levels of specific miRNAs through target-directed miRNA degradation (TDMD), further expanding the functional toolkit of NRF2.

## 2. Materials and Methods

### 2.1. Bioinformatics Analysis

The script used in this study, with some modifications, was previously described [17]. Briefly, it uses, as input, a Browser Extensible Data (BED) file containing the chromatin immunoprecipitation sequencing (ChIP-seq) peaks for the transcription factors of analysis, a text file containing a list of RefSeq transcript accession numbers, and a position frequency matrix (PFM) file from the JASPAR database containing the consensus transcription factor-binding sites to be computed. Additionally, it makes use of the BED file at the UCSC Genome Browser, Table Browser resource (https://genome.ucsc.edu/cgi-bin/hgTables) (accessed on 3 February 2023) containing the locations of every transcript and its RefSeq accession number in the genome. To identify regulatory elements, a combined segmentation BED file was generated by concatenating Combined Segmentations [18] at the UCSC Genome Browser for the hg19 human genome, using BEDTools, or the ENCODE Candidate Cis-Regulatory Elements (cCREs) [19] combined from all cell type tracks was used for the mm10 mouse genome. The script retrieves the genomic coordinates for the desired transcripts, extends them 5000 bp upstream of the transcription start site, and intersects them with ChIP-seq peaks downloaded from all experiments in ChIP-Atlas [20] for the given transcription factors using the wrapper of BEDTools for Python, pybedtools [21,22]. In this analysis, all the available binding sites for *NFE2L2*, *MAFF*, *MAFK*, *MAFG*, and *BACH1* or their mouse orthologs were downloaded and intersected with the extended transcripts of *HDAC1*, *HDAC2*, *HDAC3*, *SIRT1*, *DNMT1*, *DNMT3A*, *DNMT3B*, *DROSHA*, *DGCR8*, *DICER1*, and *TARBP2* genes or their mouse orthologs. Then, the sequences of the ChIP-seq peaks were extracted using pybedtools from the FASTA file of the hg19 human genome (Supplementary Table S1) or the mm10 mouse genome (Supplementary Table S2). The profile for *NFE2L2* was downloaded from the JASPAR database [23] in PFM format from the entry MA0150.1. Absolute frequencies were turned into a PSSM (position specific-scoring matrix), containing scores through the log2(odds-ratio) (odds ratio: observed frequency/expected frequency). One unit was added as a pseudo-count to each absolute frequency to avoid log(0). The scoring of each site followed a similar procedure as we have previously described [5]. Briefly, a sliding window of a width dependent on the profile to be used was passed over the extracted sequences. Each nucleotide in the sliding window received a score according to the PSSM, and then, the score from each nucleotide was added up in order to provide an absolute score for the site. The relative score, the maximal and minimal scores, were obtained with a given PSSM and computed as (absolute score + |minScore|)/(|maxScore| + |minScore|). Sites with a relative score below 0.8 were discarded, and the remaining ones were provided as a BED file. In order to detect active regions, the script makes use of pybedtools to intersect the segmentation file with the regions described in the regulatory element bed file.

### 2.2. Cell Cultures and Treatments

*Nfe2l2^+/+^* and *Nfe2l2^−/−^* littermate MEFs were derived from E11.5 mouse embryos and immortalized with SV40 large T antigen and provided by Dr. Antonio Cuadrado (Universidad Autónoma de Madrid, Spain) and are previously described [24,25,26]. *Keap1^−/−^* and *Keap1^+/+^* MEFs were provided by Dr. Ken Itoh (Center for Advanced Medical Research, Hirosaki University Graduate School of Medicine, Hirosaki, Japan). Mouse embryonic fibroblasts (MEFs) and mouse hippocampus-derived HT22 cells were grown in Dulbecco’s modified Eagle’s medium (D5648, Sigma-Aldrich, St. Louis, MO, USA) supplemented with 10% fetal bovine serum (CH 30160.03, HyClone, Logan, UT, USA) and 80 μg/mL gentamicin (763011.1, Laboratorios Normon, Madrid, Spain). All cell lines were mycoplasma-free, as ascertained through regular tests. Cells were changed to serum-free DMEM without antibiotics 16 h before the addition of dimethyl fumarate (DMF-20 μM) treatment (Merck, Darmstadt, Germany).

### 2.3. Analysis of mRNA Levels via Quantitative Real-Time PCR

Total RNA extraction, reverse transcription, and quantitative polymerase chain reaction (qPCR) were performed as detailed in previous articles [27]. Primer sequences are shown in Supplementary Table S3. Data were analyzed using the 2^−ΔΔCT^ method, with normalization of the raw data based on the geometric mean of *Actb* and *Gapdh* (Applied Biosystems), encoding housekeeping proteins. All PCR amplifications were performed in triplicate.

### 2.4. Plasmids

The expression vector pcDNA3.1/V5HisB-mNRF2ΔETGE was described in McMahon et al. [11]. pEF-ΔNRF2(DN), was kindly provided by Dr. J. Alam (Dept. of Molecular Genetics, Ochsner Clinic Foundation, New Orleans, LA). For luciferase assays, transient transfections were performed with the expression vector pSGG-NRF2-3’UTR, kindly provided by Dr. Qun Zhou (University of Maryland School of Medicine, Baltimore, USA), which is a reporter plasmid containing the wild-type NRF2 3’UTR.

### 2.5. Luciferase Assays

Transient transfections of MEFs or HT22 cells and luciferase assays were performed as described in [26]. pTK-Renilla was used as an internal control vector (Promega).

### 2.6. Immunoblotting

Whole-cell lysates were prepared in RIPA-Buffer (25 mM Tris-HCl pH 7.6, 150 mM NaCl, 1 mM EGTA, 1% Igepal, 1% sodium deoxycholate, 0.1% SDS, 1 mM PSMF, 1 mM Na_3_VO_4_, 1 mM NaF, 1 μg/mL aprotinin, 1 μg/mL leupeptin, and 1 μg/mL pepstatin). Whole-cell lysates were loaded for SDS-PAGE. Immunoblots were performed as described in [26]. The primary antibodies used are described in Supplementary Table S4.

### 2.7. Antisense Oligonucleotide (ASO) Pull-Down Assay

To identify *Nfe2l2* mRNA-associated miRNAs, ASO pull-down was performed using non-overlapping biotinylated ASOs recognizing LacZ (four ASOs) and Nfe2l2 (eleven ASOs). Incubation of whole-cell lysates with biotinylated ASOs was followed by incubation with Streptavidin-coupled Dynabeads™ (Invitrogen by Thermo Fisher Scientific, Waltham, MA, USA). RNAs were isolated from the pull-down materials, and qPCR was performed. Briefly, whole-cell lysates were prepared using RIPA buffer with a cocktail of protease and phosphatase inhibitors (Thermo Fischer Scientific, Waltham, MA, USA). One milligram of whole-cell extract was incubated with 1 μg of either *Nfe2l2* ASO or control LacZ ASO for 16 h at 4 °C, whereupon RNA complexes were isolated with M-280 Streptavidin Dynabeads (Invitrogen) for 2 h at 25 °C. Total RNA was isolated with Trizol-Choloroform and cDNAs were synthesized with the Maxima Reverse Transcription kit following the manufacturer’s protocols (Thermo Fisher Scientific, Waltham, MA, USA); real-time quantitative (q)PCR analysis was then performed using a PCR kit according to manufacturer’s instructions (KAPA Biosystems, Wilmington, MA, USA), and the mRNA abundance was calculated via the 2^−ΔΔCT^ analysis method, using *Gapdh* mRNA levels as the control transcript for normalization. miRs were reverse-transcribed using the MiR-X kit (Takara Bio, Shiga, Japan) and quantified via qPCR analysis using U6 as the normalization control RNA.

### 2.8. Analysis of miRNA Levels via Quantitative Real-Time PCR

Total RNA extraction was performed using the miRNeasy Mini Kit (Qiagen, Maryland, Germany). For reverse transcription and qPCR, we used miRCURY LNA SYBR^®^ Green PCR Kit (Qiagen, Maryland, Germany). We employed hsa-miR-128-3p miRCURY LNA (YP00205995), mmu-miR-155-5p miRCURY LNA (YP02119303), and miR-103a-3p miRCURY LNA (YP00204063) PCR Assays (Qiagen, Maryland, Germany). Data analyses were based on the ΔΔCT method, with normalization of the raw data based on UniSp6 (Applied Biosystems, Foster City, CA, USA). All PCR amplifications were performed from at least triplicate samples.

### 2.9. Statistical Analyses

Data are presented as the mean ± SEM. To determine the statistical test to be used, we employed GraphPad Instat 3, which includes the analysis of the data to a normal distribution via the Kolmogorov–Smirnov test. In addition, statistical assessments of differences between groups were analyzed (GraphPad Prism 8 by Dotmatics, San Diego, CA, USA) by performing an unpaired Student’s *t-*tests. A one-way ANOVA with post-hoc Newman–Keuls test was used.

## 3. Results

The epigenome is comprised of modifications to chromatin, including histone modifications and DNA methylation. One of the main histone modifications is acetylation, which is often a necessary precursor to other modifications, such as phosphorylation, methylation, and ubiquitylation. Acetylation is controlled by the opposing functions of two families of enzymes: histone acetyltransferases (HATs) and histone deacetylases (HDACs). HDACs are involved in key biological functions, such as transcription, cell cycle, autophagy, DNA damage repair, stress responses, and senescence [28,29]. HDACs are classified according to their sequence similarities with yeast HDACs into class I, class II (IIa and IIb), class III, and class IV. HDACs have been found to regulate NRF2 signaling by directly modulating NRF2 acetylation [30]. In this study, we focused on the impact of NRF2 on the expression of HDAC1, HDAC2, and HDAC3. We also tested SIRT1 (a type-IV HDAC) given that its crosstalk with NRF2 has been previously described [31]. DNA methylation, another major epigenetic change, results from the transfer of a methyl group onto the cytosine to form 5-methylcytosine. DNA methylation regulates gene expression by recruiting proteins involved in gene repression or by inhibiting the binding of transcription factor(s) to DNA [32]. DNMTs include DNMT1, with a maintenance function to copy DNA methylation patterns from the parental DNA strand onto the newly synthesized daughter strand during DNA replication, and DNMT3a and DNMT3b, with de novo functions to establish new methylation patterns on unmodified DNA. DNA methylation is crucial for regulating tissue-specific gene expression, genomic imprinting, and X chromosome inactivation. Given that oxidative stress modulates DNA methylation levels in cancer [33], cardiovascular diseases, and type 2 diabetes [34], we sought to study if the expression of HDACs and DNMTs is dependent on the transcription factor NRF2.

### 3.1. Identification of Putative ARE Sequences in HDCAs and DNMTs

First, to define comprehensively the role of NRF2 in the transcriptional regulation of HDACs and DNMTs, we searched for putative ARE sequences in the ChIP-Atlas database, an integrative database covering almost all public data archived in the Sequence Read Archive of NCBI, EBI, and DDBJ, using ChIP-seq data [20] of the human (Table 1) or mouse (Table 2) genomes for *HDAC1*, *HDAC2*, *HDAC3*, *SIRT1*, *DNMT1*, *DNMT3a*, and *DNMT3b*.

The ChIP-Atlas database includes experimental data from chromatin immunoprecipitation (ChIP) analysis of the ARE-binding transcription factors *MAFG*, *MAFF*, *MAFK*, *BACH1*, and *NFE2L2*. We used Python-based bioinformatic analysis to scan this binding region for the consensus ARE, as established in the JASPAR database [17,35]. Depending on the gene, we detected zero, one, or several putative ARE sequences with a relative score higher than 80%, a commonly used threshold for transcription factor binding-site analysis [36,37]. These putative ARE sequences in the promotor region have a high degree of similarity with the consensus ARE sequence (N**TGAC**NNN**GCN**) described by [38]. As shown in Table 1 and Table 3, bioinformatic analyses suggested that there are ARE sequences in HDACs, especially in DNMTs. We then performed functional assays to test the putative function of these sequences.

### 3.2. HDCAs Are NRF2-Dependent Genes

**Figure 1:**
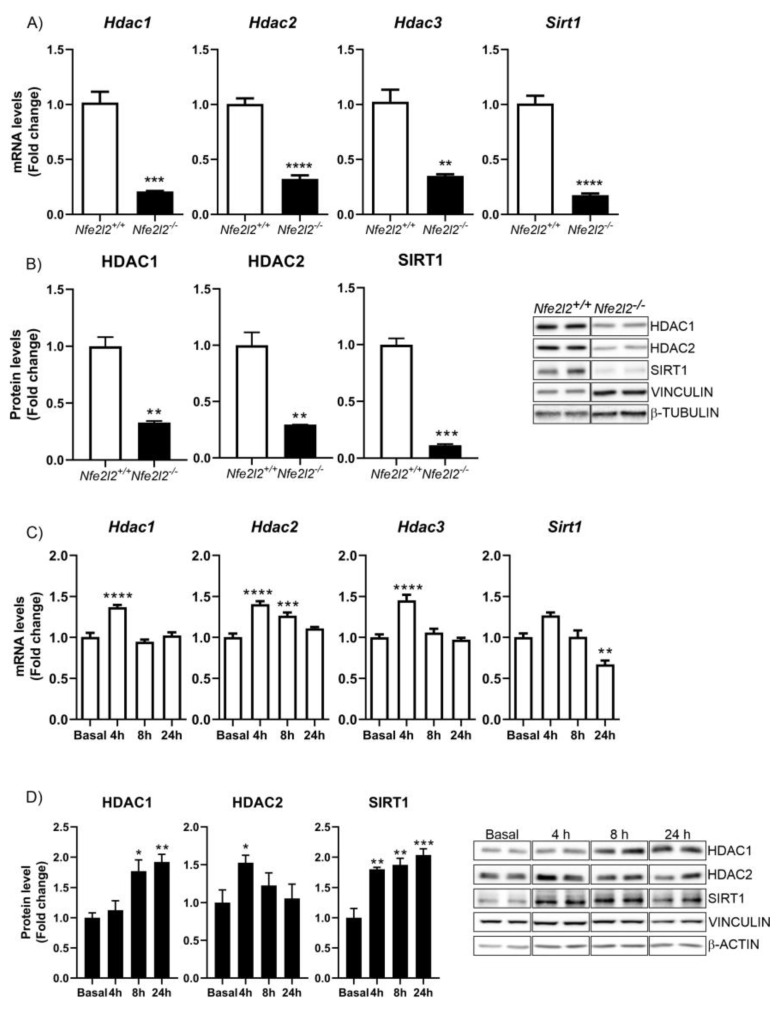
**NRF2 promotes the expression of HDCAs.** (**A**) qPCR analysis of the levels of *Hdac1*, *Hdac2*, *Hdac3*, and *Sirt1* mRNAs in *Nfe2l2^+/+^* and *Nfe2l2^−/−^* MEFs. n = 34 samples ± SEM. (**B**) The protein levels of HDAC1, HDAC2, HDAC3, and SIRT1 were analyzed via immunoblotting and their respective quantification based on densitometry using the same samples as in (**A**). n = 3–4 samples per experimental group ± SEM. Asterisks denote significant differences ** *p* < 0.01 and *** *p* < 0.001, comparing the indicated group with the wild-type mice according to a Student’s *t-*test. Time-course analysis of treatment of hippocampal HT22 cells with DMF (20 μM). (**C**) RT-qPCR analysis of the levels of *Hdac1*, *Hdac2*, *Hdac3*, and *Sirt1* mRNAs. n = 4−5 samples ± SEM. (**D**) The protein levels of HDAC1, HDAC2, HDAC3, and SIRT1 were analyzed via immunoblotting and their respective quantification based on densitometry. n = 3−4 samples per experimental group ± SEM. The one-way ANOVA test with a Newman–Keuls posterior test was used to evaluate differences in significance between groups: * *p* < 0.05, ** *p* < 0.01, *** *p* < 0.001 and **** *p* < 0.0001 compared to basal levels.

To test the bioinformatic predictions, we analyzed the levels of HDAC mRNAs in mouse embryonic fibroblasts (MEFs) derived from *Nfe2l2^+/+^* and *Nfe2l2^−/−^* mice (Supplementary Figure S1A-verification of MEF genotype). As shown, the ablation of NRF2 led to a significant decrease in mRNA levels (Figure 1A). These reductions were reflected at the protein level (Figure 1B), further supporting the view that the production of HDAC1, HDAC2, HDAC3, and SIRT1 was regulated by NRF2. We then studied whether inducing NRF2 levels might enhance HDAC expression in hippocampal HT22 neurons (Supplementary Figure S1B,C). We treated cells with dimethyl fumarate (DMF), an activator of the NRF2 pathway through both the KEAP1 and GSK-3 pathways, as previously described [39]. Treatment with DMF induced a time-dependent increase in the levels of all HDAC mRNAs (Figure 1C). These results were corroborated at the protein level, as we found that DMF treatment significantly induced protein levels of HDAC1, HDAC2, and SIRT1 (Figure 1D). Taken together, these data indicate that NRF2 is able to modulate the expression of HDAC1, HDAC2, HDAC3, and SIRT1 in different cell types, suggesting a novel function with an epigenetic impact on this transcription factor.

### 3.3. NRF2 Is a Modulator of DNMTs Expression

**Figure 2:**
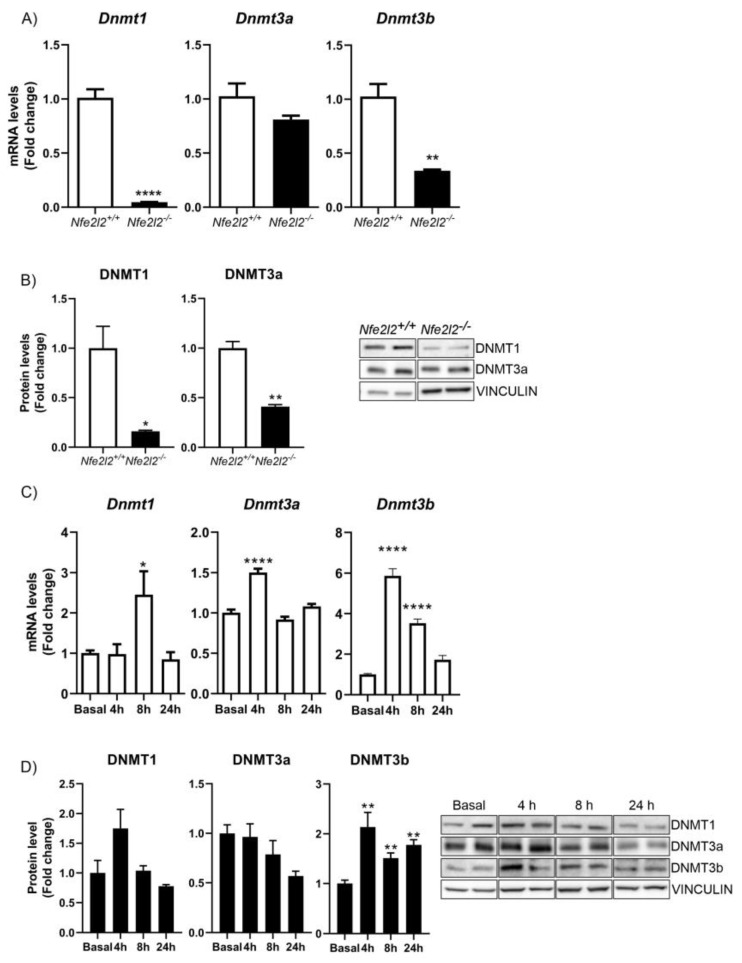
**NRF2 promotes the expression of DNMT1 and DNMT3b**. (**A**) qPCR measurement of the levels of *Dnmt1*, *Dnmt3a*, and *Dnmt3b* mRNAs in *Nfe2l2^+/+^* and *Nfe2l2^−/−^* MEFs. n = 3−4 samples ± SEM. (**B**) The protein levels of DNMT1, DNMT3a, and DNMT3b were analyzed via immunoblotting and quantification based on densitometry using the same samples as in (**A**). n = 3−4 samples per experimental group ± SEM. Asterisks denote significant differences: * *p* < 0.05, ** *p* < 0.01, and **** *p* < 0.0001, comparing the indicated group with the wild-type mice according to a Student’s *t-*test. Time-course analysis of treatment of hippocampal HT22 cells with DMF (20 μM). (**C**) RT-qPCR analysis of the levels of *Dnmt1*, *Dnmt3a*, and *Dnmt3b* mRNAs. n = 4−5 samples ± SEM. (**D**) Protein levels of DNMT1, DNMT3a, and DNMT3b were analyzed via immunoblotting and respective quantification based on densitometry. n = 3−4 samples per experimental group ± SEM. The one-way ANOVA test with a Newman–Keuls posterior test was used to evaluate differences in significance between groups: ** *p* < 0.01 compared to basal levels.

After bioinformatic analysis of ARE elements in the promoters of DNMTs, we followed the same strategy for the analysis of HDACs. First, we analyzed the levels of *Dnmt1*, *Dnmt3a*, and *Dnmt3b* mRNAs in NRF2-deficient MEFs. We observed that the levels of *Dnmt1* and *Dnmt3b* mRNAs were significantly reduced, while the changes in *Dnmt3a* mRNA were more modest (*p* = 0.07) (Figure 2A). These changes were mirrored at the protein level for DNMT1; DNMT3a levels were moderately reduced, while DNMT3b was not detectable (see Figure 2B). We then analyzed the effects of inducing NRF2 by treating HT22 cells with DMF. As shown, DMF treatment induced DNMT1 and DNMT3b at both mRNA and protein levels, validating the effects observed in MEFs and further supporting the notion that they are regulated by NRF2. For DNTM3a, there was a slight induction of mRNA levels but not at the protein level (Figure 2C,D); further analysis is needed to determine whether the expression of DNMT3a is regulated by NRF2.

### 3.4. NRF2 Regulates the Expression of Proteins Implicated in miRNA Biogenesis

Besides histone acetylation and DNA methylation, miRNAs (small non-coding RNAs) reduce the stability and translation of target mRNAs and are also epigenetic regulators. ROS can modulate miRNA biogenesis at many levels, and several enzymes and components of the miRNA processing machinery can be affected by ROS [40]. Therefore, we examined the possibility that expression of the proteins involved in microRNA biogenesis (DGCR8, DROSHA, DICER, and TARBP2) might be regulated by NRF2.

Therefore, as before, we investigated bioinformatically whether there were ARE sequences in the promoter regions of the *DGCR8*, *DROSHA*, *DICER1*, and *TARBP2* genes. We observed that, especially in the human genome (Table 3), there were several possible ARE sequences within all genes analyzed. In the mouse genome, we only found possible ARE sequences in the regulatory regions of the *Dicer1* and *Drosha* genes (Table 4).

**Figure 3:**
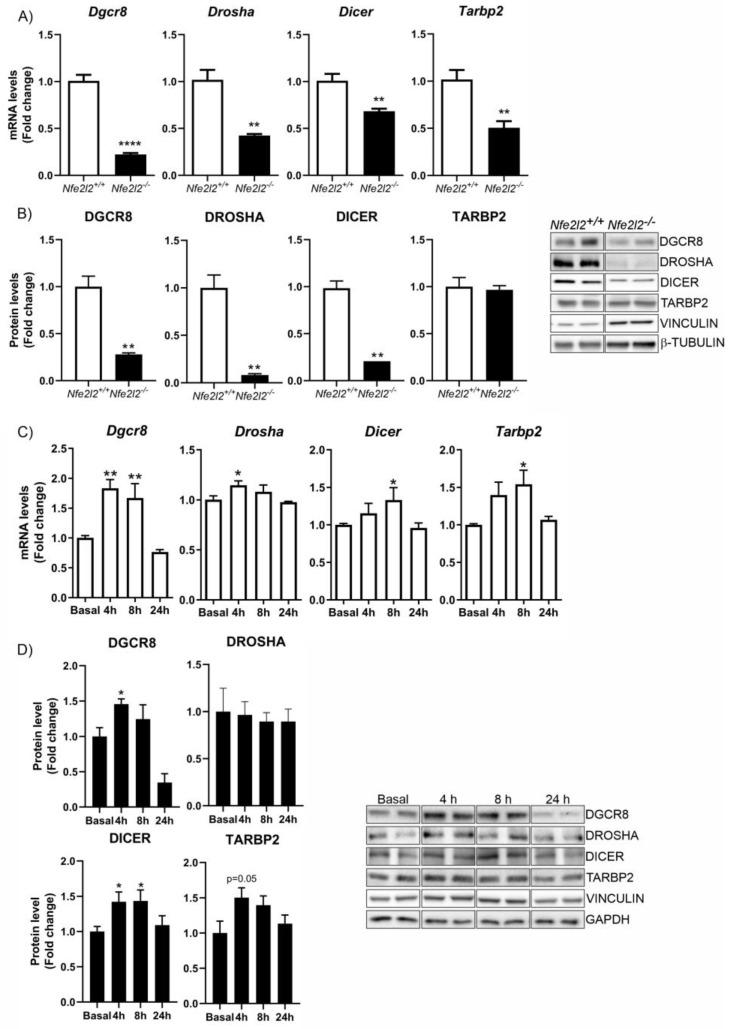
**Impact of NRF2 on the expression levels of several proteins implicated in miRNA biogenesis.** (**A**) qPCR analysis of the levels of *Dgcr8*, *Drosha*, *Dicer1*, and *Tarbp2* mRNAs n = 3−4 samples ± SEM. (**B**) In the same cells described in (**A**), the levels of DGCR8, DROSHA, DICER, and TARBP2 proteins were analyzed via immunoblotting and quantification based on densitometry using the same samples as in (**A**). n = 3−4 samples per experimental group ± SEM. Asterisks denote significant differences: ** *p* < 0.01 and **** p < 0.0001, comparing the indicated group with the wild-type mice according to a Student’s *t-*test. Time-course analysis of treatment of hippocampal HT22 cells with DMF (20 μM). (**C**) qPCR analysis of the levels of *Dgcr8*, *Drosha*, *Dicer1*, and *Tarbp2* mRNAs. n = 4−5 samples ± SEM. (**D**) In the cells described in (**C**), the levels of DGCR8, DROSHA, DICER, and TARBP2 were analyzed via immunoblotting and respective quantification based on densitometry. n = 3−4 samples per experimental group ± SEM. The one-way ANOVA test with a Newman–Keuls posterior test was used to evaluate differences in significance between groups: * *p* < 0.05 and ** *p* < 0.01 compared to basal levels.

In MEFs from *Nfe2l2^+/+^* and *Nfe2l2^−/−^* mice, we observed significant decreases in *Dgcr8*, *Drosha*, *Dicer1*, and *Tarbp2* mRNAs (Figure 3A). Similarly, the expression levels of DGCR8, DROSHA, and DICER1 proteins decreased greatly in the absence of NRF2 (Figure 3B), while the levels of TARDBP2 protein did not change. On the other hand, treatment of HT22 cells with DMF significantly induced the levels of *Dgcr8*, *Drosha*, *Dicer1*, and *Tarbp2* mRNAs (Figure 3C), as well as the proteins DGCR8 and DICER, while TARBP2 was less induced and DROSHA was unchanged (Figure 3D). These data suggest that many proteins involved in miRNA biogenesis are regulated by NRF2.

### 3.5. NRF2 Modulates miRNA Expression

**Figure 4:**
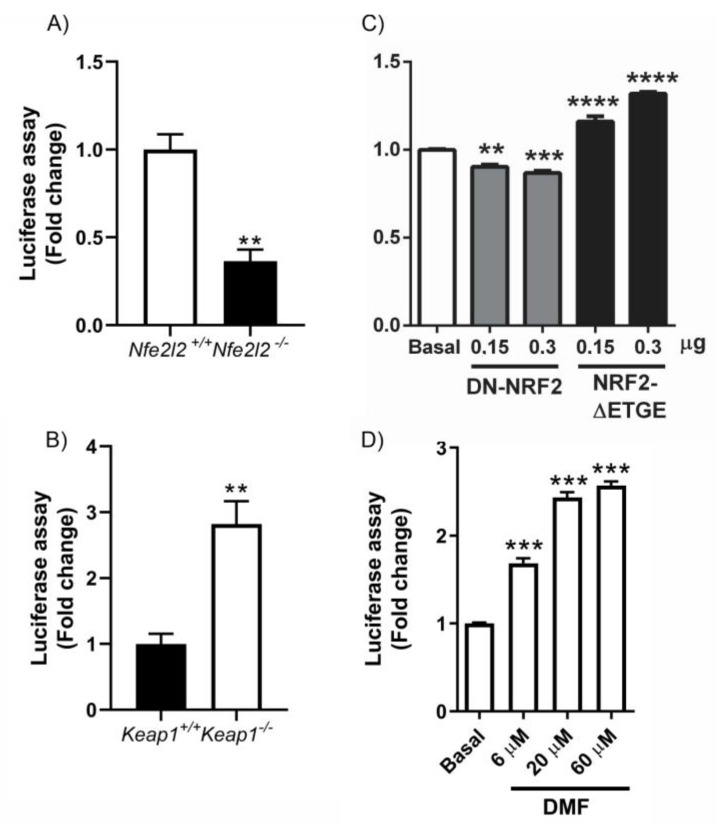
**Function of the 3’UTR-*Nfe2l2* via luciferase reporter analysis.** The potential ability of miRNAs or other factors to regulate the 3’ untranslated region (3’UTR) of *Nfe2l2* mRNA was evaluated using a luciferase reporter. (**A**) *Nfe2l2^+/+^* and *Nfe2l2^−/−^* MEFs cells and (**B**) *Keap1^−/−^* and *Keap1^+/+^* MEF cells were transfected with pSGG-NRF2-3’UTR. (**C**) HT22 cells were transfected with pSGG-NRF2-3’UTR, pEF-ΔNRF2(DN), or pcDNA3.1/V5HisB-mNRF2ΔETGE, respectively. Asterisks denote significant differences: ** *p* < 0.01, *** *p* < 0.001 and **** p < 0.0001, comparing the indicated group with the control group according to a Student’s *t-*test. (**D**) HT22 cells were transfected with pSGG-NRF2-3’UTR and treated with DMF at 6 μM, 20 μM, or 60 μM for 16h. All luciferase activities were measured 24 h after transfection. The luciferase activities were normalized to the renilla luciferase activities from the co-transfected reporter. The relative luciferase activities were calculated by normalizing them to those of controls. n = 3 samples per experimental group ± SEM. The one-way ANOVA test with a Newman–Keuls posterior test was used to evaluate differences in significance between groups: ** *p* < 0.01 and *** *p* < 0.001 compared to basal levels.

The sequences of 3′-untranslated regions (3’UTRs) of messenger RNAs (mRNAs) govern their stability, localization, and expression [41]. miRNAs typically bind to the 3′ UTRs of target mRNAs with which they share partial complementarity and reduce their stability and translation. Therefore, we evaluated whether miRNAs interacting with the 3’UTR of NFE2L2 mRNA suppressed NRF2 expression and whether NRF2 was able to modulate this loop. To test this hypothesis, the ability of miRNAs to regulate the 3’UTR of Nfe2l2 mRNA was evaluated using luciferase reporters. MEFs from Nfe2l2^+/+^ and Nfe2l2^−/−^ mice were transfected with the pSGG luciferase vectors bearing the NRF2 3’UTR, along with a control renilla vector. As shown in Figure 4A, the absence of NRF2 led to increased expression of miRNAs that negatively modulate the expression of the 3’UTR. Similarly, these data were confirmed in HT22 cells, where transfection of a dominant negative NRF2 (DN-NRF2) negatively regulated the expression of the 3’UTR of NRF2 (Figure 4C). In contrast, an increase in NRF2 levels, either in KEAP1-deficient MEFs (where there is an increase in NRF2 levels) (Figure 4B) or via transfection of the NRF2-ΔETGE-V5 (Figure 4C) that lacks four residues (ETGE) essential for recognition by the E3 ligase complex Cul3/KEAP1, led to a decrease in the levels of miRNAs that inhibit the 3’UTR of NRF2 and thus an increase in its expression. These data were verified using the NRF2 inducer DMF at different doses (Figure 4D). The results indicate a dose–response effect in which the higher the increase in NRF2 levels the greater the de-repression of the reporter 3’UTR. Although in these experiments we cannot rule out the impact of other factors, such as RNA-binding proteins, other non-coding RNAs, or RNA modifications on this 3’UTR, in addition to the action of miRNAs, the data are consistent with a model whereby NRF2 reduces the levels of specific microRNAs capable of binding the Nfe2l2 mRNA and reducing Nfe2l2 mRNA and NRF2 protein.

### 3.6. Implication of NRF2 Expression in Target-Dependent miRNA Degradation (TDMD) of miR-155-5p

**Figure 5:**
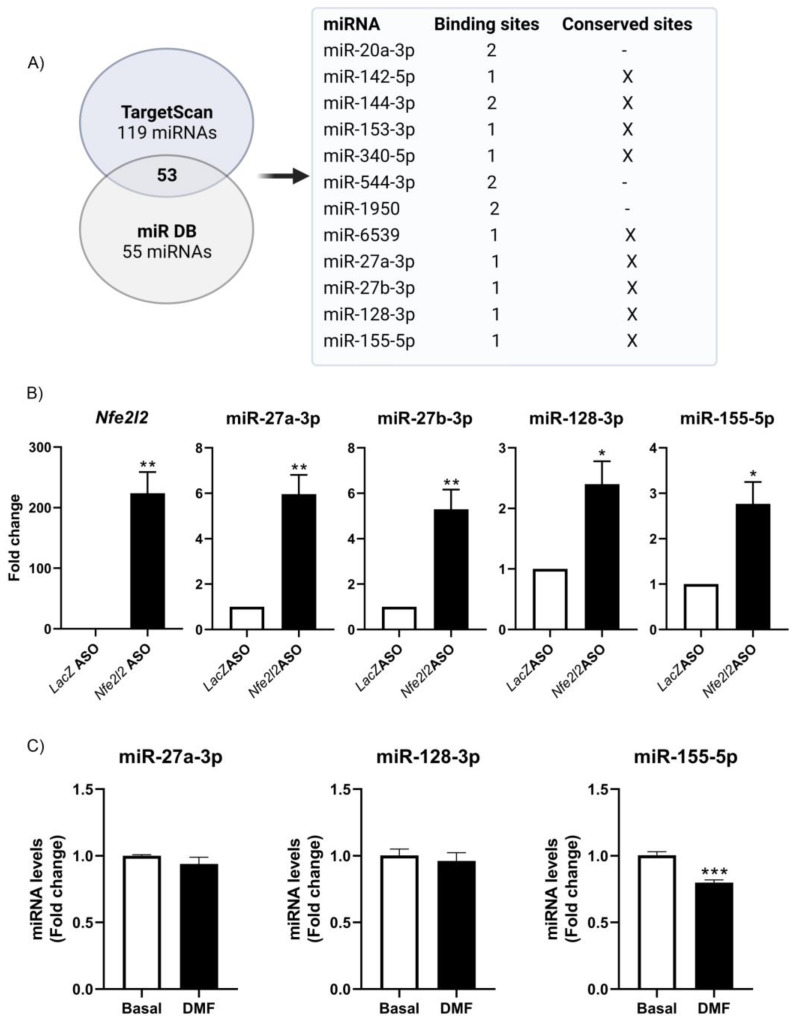
**Association of miR-27a-3p, miR-27b-3p, miR-128-3p and miR-155-5p with Nfe2l2 mRNA and possible role of NRF2 mRNA in TDMD of miR-155-5p.** (**A**) Study design. Of the 53 miRNAs common between both databases, we examined which ones contained either two binding sites or conserved sites or both. These 12 miRNAs were further analyzed. (**B**) Biotinylated antisense oligomers (ASOs) complementary to the *Nfe2l2* mRNA and LacZ were incubated with HT22 lysates. After affinity pulldown using streptavidin beads, the levels of miRNA enrichment in the ASO-pulldown samples were assessed via RT-qPCR analysis, and only miR-27a-3p, miR-27b-3p, miR-128-3p, and miR-155-5p were enriched (relative to the enrichment of *Gapdh* mRNA, a transcript that does not bind the ASOs and encodes a housekeeping protein) in the pulldown samples. (**C**) HT22 cells were treated with DMF (20 μM) for 16 h, and miRNA levels were measured via qPCR analysis. Asterisks denote a significant difference: * *p* < 0.05, ** *p* < 0.01, and *** *p* < 0.001 compared to the corresponding control group according to a Student’s *t-*test.

The fact that NRF2 expression levels could modulate the levels of miRNAs led us to identify the specific miRNAs involved. We first analyzed which miRNAs might be binding to the *Nfe2l2* mRNA, by using the Targetscan (119 miRNAs) and miR DB (55 miRNAs) databases (Figure 5A). In total, there were 53 merged miRNAs. Of these 53 miRNAs, there were miRNAs with two binding sites (miR-144-4p, miR-1950, miR-20a-3p, and miR-544-3p) and with conserved sites (miR-27b-3p, miR-27a-3p, miR-6539, miR-128-3p, miR-142a-5p, miR-340-5p, miR-153-3p, miR-155-5p, and miR-144-3p). To investigate these interactions directly, 11 antisense oligonucleotides (ASOs) directed at *Nfe2l2* mRNA (NM_010902.4) were designed in order to pull down the endogenous *Nfe2l2* mRNA. Whole-cell lysates from HT22 cells were then prepared and incubated with either *Nfe2l2* ASOs or control LacZ ASO, whereupon RNA complexes were isolated with streptavidin Dynabeads (Invitrogen). As shown in Figure 5A, *Nfe2l2* mRNA was highly enriched in *Nfe2l2* ASO samples compared to that in LacZ ASO samples. We then measured the levels of all the predicted miRNAs (Figure 5A) in the pulldown complexes and found that miR-27a-3p, miR-27b-3p, miR-128-3p, and miR-155-5p were enriched in *Nfe2l2* ASO-pulldown samples (Figure 5B), suggesting that these miRNAs are associated with *Nfe2l2* mRNA.

**Figure 6:**
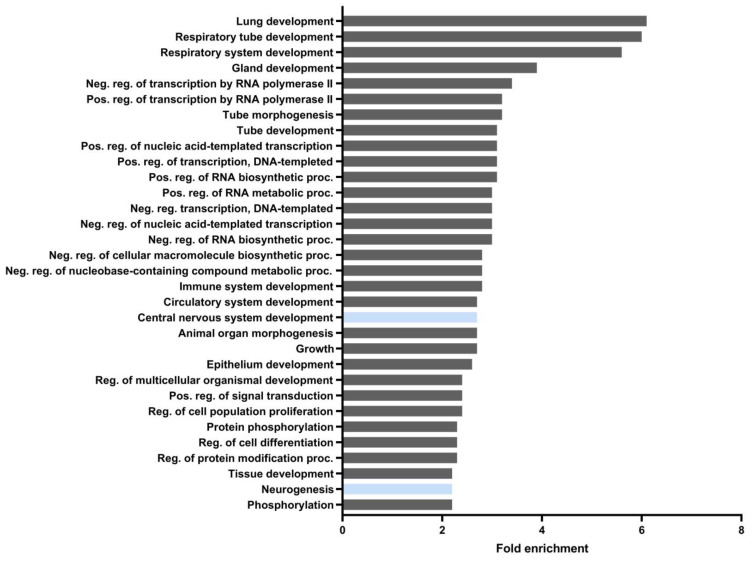
**GO biological process analysis using the ShinyGo 0.76.2 platform.** Neuronal-related functions are highlighted (blue). Using the TargetScan platform, the list of genes that can be modulated by miR-155-5p was extracted. These genes were analyzed using the ShinyGO 0.76.2 pathway database GO biological process platform.

As mentioned above, miRNAs control target gene expression by inhibiting translation and degrading target RNAs. In addition, there is evidence that mRNAs can affect microRNA activity in two manners [42]. First, some mRNAs can function as competing endogenous RNAs (ceRNAs), as seen when two endogenous targets compete with each other for binding to a shared miRNA [43]. In this case, if one of the endogenous mRNA targets changes its expression, the activity of miRNAs upon the other targets will change accordingly. This mechanism appears to have been ruled out, since NRF2 overexpression itself reduced the levels of the miRNAs that bind to it. The second mechanism is target-directed miRNA degradation (TDMD) [44]. In TDMD, the target mRNA promotes the degradation of the miRNA that binds to it [45]. To test whether this might be what was happening with NRF2, we treated HT22 cells with DMF (conditions similar to Figure 4D, 20 μM) and analyzed the expression levels of miR-27a-3p, miR-128-3p, and miR-155-5p. As observed in Figure 5C, NRF2 induction promoted a significant decrease in miR-155-5p levels, while other miRNAs were unchanged. Finally, we sought to determine in which biological processes miR-155-5p is involved, mainly at the neuronal level, in order to determine the role that its modulation through NRF2 may have. To do this, we determined its targets through the TargetScan database, and using the ShinyGo 0.76.2 platform and its pathway database, we performed a GO biological process analysis. Although miR-155-5p participates in many processes, it was found to be involved in the regulation of ~45 mRNAs encoding proteins that participate in the development of the central nervous system (CNS) and ~64 mRNAs encoding proteins implicated in neurogenesis (Figure 6), respectively. Thus, a more exhaustive analysis of the involvement of miR-155-5p and the possible regulation by NRF2 in the development of the central nervous system is required.

## 4. Discussion

Although the expression of the transcription factor NRF2 at the epigenetic level has been studied extensively, the reciprocal process, i.e., the impact of NRF2 on epigenetic gene regulation, has not been explored. In this study, we show for the first time that NRF2 is able to regulate the expression of type-I HDACs (HDAC1, HDAC2, HDAC3) and SIRT1, DNMTs, and proteins involved in miRNA biogenesis, DROSHA, DGCR8, DICER1, and TARBP2. Our data further suggest that NRF2 may be involved in the regulation of the levels of certain miRNAs through TDMD. These new data are of great potential relevance to the pharmacological regulation of NRF2 as a therapeutic strategy for various pathologies since epigenetic changes triggered by NRF2 will also have to be weighed.

**Figure 7:**
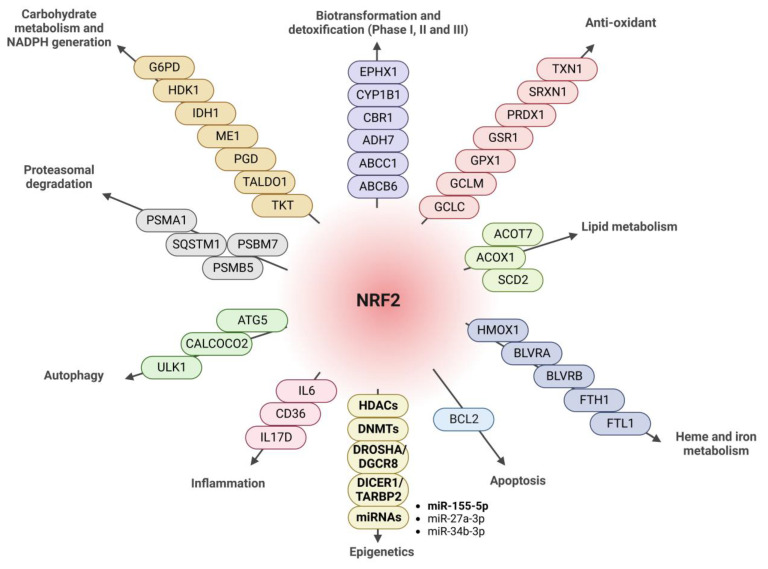
Diagram of the main targets of NRF2. NRF2 is implicated in the regulation of biotransformation and detoxification proteins (Phase I, II, III). ABCB6: ATP-binding cassette, subfamily B (MDR/Tap) member 6; ABCC1: ATP-binding cassette, subfamily C (CFTR/MRP); ADH7: alcohol dehydrogenase class 4 mu/sigma chain; CBR1: carbonyl reductase 1; CYP1B1: cytochrome P450; EPHX1 epoxide hydrolase 1, microsomal. Antioxidants: GCLC: glutamate–cysteine ligase, catalytic subunit; GCLM: glutamate–cysteine ligase, modifier subunit; GPX1: glutathione peroxidase 1; GSR1: glutathione reductase 1; PRDX1: peroxiredoxin 1; SRXN1: sulfiredoxin 1; TXN1: thioredoxin. Lipid metabolism: ACOT7: acetyl-CoA thioesterase 7; ACOX1: acetyl-CoA oxidase 1; SCD2: stearoyl-CoA desaturase-2. Heme and iron metabolism: HMOX1: heme oxygenase 1; BLVRA: biliverdin reductase A; BLVRB: biliverdin reductase B; FTH1: ferritin, heavy polypeptide; FTL1: ferritin, light polypeptide. Apoptosis: BCL2 B:-cell lymphoma 2. Epigenetics: Type-I HDACs (HDAC1, HDAC2, HDAC3, and SIRT1); DNMTs (DNMT1, DNMT3a, and DNMT3b); DROSHA, DGCR8, DICER1, and TARBP2; miRNAs associated with NRF2: miR-155-5p, miR27a-3p, and miR-34b-3p. Inflammation: IL6: interleukin 6; CD36: cluster of differentiation 36; IL17D: interleukin-17D. Autophagy: ATG5: autophagy protein 5; CALCOCO2: calcium-binding and coiled-coil domain-containing protein 2; ULK1: Unc-51-like kinase 1. Proteasomal degradation: PSMB5: proteasome subunit beta type-5; PSMB7: proteasome subunit beta type-7; SQSTM1: sequestosome 1 (p62); PSMA1: proteasome 20S subunit alpha 1. Carbohydrate metabolism and NADPH generation: TKT: transketolase isoform 1; TALDO1: transaldolase; PGD: 6-phosphogluconate dehydrogenase; ME1: malic enzyme 1; IDH1: NADP-dependent isocitrate dehydrogenase; HDK1: hexokinase domain-containing 1; G6PD: glucose-6-phosphate dehydrogenase. Modified from [46] and highlighting the new NRF2 targets described in this study.

NRF2 is a pleiotropic transcription factor capable of regulating the expression of genes involved in different processes, including xenobiotic, redox, and carbohydrate metabolism, inflammation, and proteostasis [5] (Figure 7). Therefore, its dysregulation has been described in a multitude of pathologies, in many cases along with epigenetic changes that cause aberrant gene expression programs and the loss of homeostasis. These data suggest that NRF2 affects many cellular functions underlying disease, although to-date, we only understand how epigenetic modifications affect the expression and function of the NRF2 pathway. The fact that NRF2 can promote the expression of type-I HDAcs (Figure 1), DNMTs (Figure 2), and proteins involved in miRNA biogenesis (Figure 3) opens new perspectives on the spectrum of actions of NRF2, its epigenetic influence, and its implications in disease. In this study, we have focused on the study of type-I and SIRT1 HDACs, but in further studies, it will be interesting to determine the involvement of NRF2 related to the other HDACs and to determine whether the results of the study presented here can be extrapolated to all types of HDACs or is specific to type-I HDACs and SIRT1.

Our studies suggest that when NRF2 levels are decreased in pathologies, this reduction may lead to decreased expression of Type-I HDACs and increased expression of genes that were repressed under physiologic conditions. Similarly, NRF2 deficiency would also lower DNMT levels, in turn inducing the expression of genes that were previously repressed. On the other hand, NRF2 inducers, such as sulforaphane, were found to induce NRF2 regulation at the epigenetic level, mainly associated with DNA methylation [47]. Epigenetic changes in the mechanism of action of DMF have also been described [48,49,50]. In contrast with the several agents that function as NRF2 inducers, very few molecular components have been recognized as NRF2 inhibitors. Brusatol, luteolin, trigonelline, and retinoic acid are several compounds that have been described as having inhibitory effects on NRF2 signaling [51], but their effects at the epigenetic modulation level have not yet been described. In future experiments, it will be interesting to analyze the effect of NRF2 inhibitors on the levels of HDACs, DNMTs, and miRNAs, to determine the possible impact of NRF2 inhibitor treatments at the epigenetic level. Our data support the fact that the pharmacological regulation of NRF2 involves different downstream effectors (Figure 7), including HDACs, DNMTs, and miRNAs, and thus broadening the spectrum of action of this transcription factor.

The results presented in this study can be potentially relevant to a wide range of pathologies where epigenetic mechanisms are of particular importance, such as cancer [52,53], allergies [54]**,** and neurodegenerative diseases [55,56]. In cancer, specific methylation and other alterations of the *NFE2L2* promoter have been documented [12,57,58], which can alter the expression levels of NRF2, linked to carcinogenesis through metabolic reprogramming, tumor promotion, inflammation, and resistance to therapy. NRF2 silencing or pharmacological inhibition by brusatol reduced the proliferation and migration of breast cancer (BC) cells, inhibited proliferation, activated apoptosis, sensitized BC cells to cisplatin in vitro, and slowed tumor cell growth in vivo [59]. Along these lines, esophageal adenocarcinoma (EAC) displayed increased NRF2, and both the knockdown of NRF2 and pharmacological inhibition with brusatol inhibited tumor growth by inducing ferroptosis and apoptosis [60]. In the case of metastatic Ewing sarcoma and osteosarcoma, it has been described that oxidative stress attenuates metastasis; here, treatment with the class-I HDAC inhibitor MS-275 inhibited the deacetylation of YB-1 (Y-box binding protein 1), reduced its binding to the 5’UTR of *NFE2L2*, reduced the translation of NRF2, and increased the levels of intracellular ROS [61]. However, other studies showed that HDAC inhibitors increased NRF2-signaling in tumor cells [62]. Therefore, further studies will be needed to look at the crosstalk between HDACs and NRF2, taking into consideration that NRF2 has pleiotropic roles in cancer cells [51].

Outside of cancer, type I and II HDAC inhibition mediated by Trichostatin A (TSA) activated transcription factor NRF2 and protected against cerebral ischemic damage. On the other hand, SIRT1 activation was also found to induce the NRF2 signaling pathway, with beneficial effects in focal cerebral ischemia [63], indicating that further studies are needed to unravel the crosstalk between HDAC and NRF2 in this pathology [64]. In neurodegenerative diseases, HDAC inhibitors were found to improve the redox balance and attenuate neuronal degeneration in Huntington’s disease [65] and amyotrophic lateral sclerosis [66] mouse models and in Alzheimer’s disease-like pathological changes in SH-SY5Y neuroblastoma cells [67]. One mechanism of action described in this regard is that HDACs remove acetyl groups in histones associated with the KEAP1 promoter region, inducing an increase in KEAP1 transcription, and therefore, the inhibition of HDAC might have opposite effects [65]. Additionally, NRF2 levels were found to be elevated in many neurodegenerative diseases [27,68,69], and thus, further studies are warranted to fully understand the interaction between NRF2 and HDACs and the therapeutic value of interventions directed at NRF2 and/or HDACs. Similar to HDACs, DNMTs are also potential therapeutic targets, since alterations in their activity have also been associated with various pathologies. Therefore, as with HDACs, more studies are needed to establish the value of therapeutic strategies that modulate NRF2 and DNMTs pharmacologically [70,71,72].

A multitude of miRNAs are predicted to repress NRF2 production [73], and many miRNAs are involved in oxidative stress processes associated with physiological and pathological conditions [74]. The fact that oxidative stress is capable of regulating the expression of proteins involved in the biogenesis of miRNAs [75,76,77] led us to evaluate the potential involvement of NRF2 in this mechanism. Our data indicate that NRF2 promotes the production of proteins DROSHA, DGCR8, DICER1, and TARBP2 involved in miRNA biosynthesis (Figure 3), expanding the function of this transcription factor into the post-transcriptional space. Although miRNAs typically modulate the stability and translation of their target mRNAs, we have shown that the absence of NRF2 leads to the increased expression of miRNAs that negatively modulate the expression of the 3’UTR-*Nfe2l2* (Figure 4A,C) and vice versa, underscoring the fact that NRF2 levels can also repress the actions of some miRNAs. More detailed studies are necessary to elucidate the specific miRNAs involved in this mechanism. As mentioned above, we cannot rule out the impact of other factors, such as RNA-binding proteins, other non-coding RNAs, or RNA modifications on this 3’UTR, in addition to the action of miRNAs. Further studies of additional regulatory factors should also be considered.

Oxidative stress is one of the main inducers of the NRF2 pathway, and thus, its activation is linked to the induction of oxidative stress-associated miRNAs, the so-called “redoximiRs”, such as miR-27a-3p and miR-155-5p [75,78]. In HT22 hippocampal cells, our data indicate that of all miRNAs analyzed (Figure 5A), only miR-27a-3p, miR-27b-3p, miR-128-3p, and miR-155-5p associate with *Nfe2l2* mRNA (Figure 5B). There is previous evidence that miR-27a-3p and miR-27b-3p are redox-sensitive miRNAs [79,80,81,82] and modulate NRF2 levels, in agreement with our results. For example, in maternal diabetes-induced oxidative stress, miR-27a-3p levels rise, in turn suppressing NRF2 production [81]. Furthermore, an analysis of miRNA signatures in transgenic mice expressing a constitutively active, cardiac-specific NRF2 (caNrf2-Tg) [15] revealed that increasing the levels of NRF2 leads to reduced miR-155-5p levels. These results support our data that the induction of NRF2 decreased miR-155-5p levels (Figure 5B). Here, we focused on “redoximiRs” and found that *NFE2L2* mRNA might drive the degradation of specific miRNAs mediated by TDMD and thereby reduce the levels of specific miRNAs. Further experiments are necessary to determine the exact mechanism by which NRF2 causes the degradation of other microRNAs, such as miR-155-5p, given its implication in neuroinflammation and other pathologies, and is the main miRNA induced by LPS treatment in microglia [83,84]. Furthermore, miR-155 alters the expression of genes that regulate axon growth [85], supporting the bioinformatic prediction that miR-155 can regulate the expression of genes involved in CNS development and neurogenesis (Figure 6). Therefore, the modulation of this miR-155-5p could be of great relevance in relation to neurodegenerative diseases, such as Alzheimer’s disease. The precise regulation of miR-155 and other microRNAs by NRF2 awaits future study. It would also be interesting to determine, in future experiments, whether these miRNAs are specific to a specific neuronal type (e.g.**,** hippocampal neurons) or have a more general functions, as well as whether there are differences between the various brain cell types.

## 5. Conclusions

This study demonstrates for the first time that the molecules involved in epigenetic gene regulation, namely HDACs, DNMTs, and miRNA biogenesis factors, are under the control by NRF2, expanding the field of action of this transcription factor.

## Figures and Tables

**Table 1:** **Table showing the putative ARE sequences identified in the human genome with a relative score over 80%.** Columns 1 and 2 describe the genes containing the transcription factor-binding sites and their specific locations according to the GRCh37 (hg19) human genome assembly. The Motif column contains the specific sequence that was identified as a putative ARE sequence. The TGA motif in nucleotides 2, 3, and 4 of the motifs and the GC motif in nucleotides 9 and 10 of the motif are in bold, if present. Relative scores were calculated against the consensus binding sequence according to the position frequency matrix of the JASPAR database. The strand column indicates in which strand of the DNA, relative to the transcript sense is the putative ARE sequence: sense (+) or antisense (-). The Regulatory Element column provides the segmentation annotation from the Combined Segmentation Track at the UCSC Genome Browser. The TFs column indicates the transcription factor for which the ChIP-seq site belonged in the ChIP-Atlas database. * TSS, transcription start site; T, transcribed region; R; repressive or low activity region; PF, promoter flanking region; E; enhancer; WE, weak enhancer; CTCF, CTCF-enriched region.

*Gene*	Coordinates (hg19 Genome)	Motif	Relative Score	Strand	Regulatory Element *	TFs
*HDAC1*	chr1:32757324-32757335	G**T**C**AC**TCA**GC**C	0.835	-	TSS	*MAFF*
*DNMT1*	chr19:10266742-10266753	A**TGAC**TTG**GC**C	0.855	+	T	*MAFK*
*DNMT1*	chr19:10266727-10266738	A**TGAC**TGA**G**GA	0.836	-	T	*MAFK*
*DNMT1*	chr19:10284277-10284288	C**TGAC**TCA**GC**C	0.883	+	T	*NFE2L2*
*DNMT1*	chr19:10288124-10288135	A**T**T**AC**TAA**GC**T	0.827	+	T	*MAFK*
*DNMT1*	chr19:10288156-10288167	AGG**AC**TAA**GC**A	0.874	+	T	*MAFK*
*DNMT3A*	chr2:25473227-25473238	C**TGAC**TCAA**C**A	0.836	+	TSS, R, PF	*MAFK*
*DNMT3A*	chr2:25524509-25524520	C**TGAC**TCA**GC**T	0.869	+	R, E	*MAFK, NFE2L2, BACH1*
*DNMT3A*	chr2:25524530-25524541	A**TGAC**TAAT**C**C	0.816	+	R, E	*MAFK, NFE2L2, BACH1*
*DNMT3A*	chr2:25524581-25524592	C**TGAC**CCT**GC**A	0.833	+	R, E	*MAFK, NFE2L2, BACH1*
*DNMT3A*	chr2:25565466-25565477	C**TG**C**C**TCA**GC**A	0.836	+	TSS, CTCF	*MAFK, MAFF*

**Table 2:** **Table showing the putative ARE sequences identified in the mouse genome with a relative score over 80%**. Columns 1 and 2 describe the genes containing the transcription factor-binding sites and their specific locations according to the GRCm38 (mm10) mouse genome assembly. The Motif column contains the specific sequence that was identified as a putative ARE sequence. The TGA motif in nucleotides 2, 3, and 4 of the motifs and the GC motif in nucleotides 9 and 10 of the motif are in bold, if present. Relative scores were calculated against the consensus binding sequence according to the position frequency matrix of the JASPAR database. The strand column indicates in which strand of the DNA, relative to the transcript sense is the putative ARE sequence: sense (+) or antisense (-). The Regulatory Element column provides the segmentation annotation from the ENCODE Candidate Cis-Regulatory Elements (cCREs) combined from all cell types of Track at the UCSC Genome Browser. The TFs column indicates the transcription factor for which the ChIP-seq site belonged in the ChIP-Atlas database. *, PLS, promoter-like signature; dELS; distal enhancer-like signature.

*Gene*	Coordinates (mm10 Genome)	Motif	Relative Score	Strand	Regulatory Element *	TFs
*Hdac2*	chr10:37001063-37001074	A**TGA**GTCA**GC**A	0.92	-	dELS	*Mafk*
*Hdac2*	chr10:37000993-37001004	A**TGA**TTG**G**GCA	0.82	-	dELS	*Mafk*
*Hdac3*	chr18:37949598-37949609	A**TGAC**TCA**GC**T	0.93	+	None	*Mafk*
*Dnmt1*	chr9:20943516-20943527	C**TG**C**C**ACA**GC**A	0.82	+	None	*Maff*
*Dnmt1*	chr9:20946724-20946735	A**TGAC**TCA**GC**A	1.00	+	None	*Mafk, Mafg*
*Dnmt1*	chr9:20952657-20952668	A**TG**C**C**TCG**GC**A	0.83	+	PLS	*Mafk, Bach1*
*Dnmt1*	chr9:20953035-20953046	G**TG**G**C**TCG**GC**A	0.83	+	PLS	*Nfe2l2, Mafk*
*Dnmt1*	chr9:20961672-20961683	G**TGAC**TCA**G**TC	0.83	-	dELS	*Maff, Mafk, Bach1*
*Dnmt3a*	chr12:3811310-3811321	T**TGAC**TCA**GC**G	0.86	+	None	*Maff*
*Dnmt3a*	chr12:3846390-3846401	T**TGAC**TCA**GC**A	0.92	-	None	*Mafk*
*Dnmt3a*	chr12:3846370-3846381	A**TGAC**TAAC**C**A	0.87	-	None	*Mafk*
*Dnmt3a*	chr12:3846273-3846284	A**TG**G**C**TTT**GC**A	0.81	-	None	*Mafk*
*Dnmt3a*	chr12:3846068-3846079	G**TGAC**CAAT**C**A	0.82	-	None	*Mafk*
*Dnmt3a*	chr12:3854616-3854627	A**TGA**GATT**GC**A	0.82	-	dELS	*Bach1*
*Dnmt3a*	chr12:3856263-3856274	A**T**A**AC**CCA**GC**A	0.85	+	dELS	*Mafk, Bach1*
*Dnmt3a*	chr12:3890847-3890858	C**TG**T**C**TCA**GC**A	0.84	-	None	*Makf*
*Dnmt3b*	chr2:153671696-153671707	A**TGA**ACCA**GC**A	0.85	+	None	*Mafk*

**Table 3:** Table showing the putative ARE sequences identified in the human genome with a relative score over 80%. Columns and content follow the same format as Table 1.

*Gene*	Coordinates (hg19 Genome)	Motif	Relative Score	Strand	Regulatory. Element	TFs
*DGCR8*	chr22:20078164-20078175	A**TGAC**TCA**G**TG	0.837	+	T	*NFE2L2*
*DGCR8*	chr22:20078089-20078100	C**TGA**AAAA**GC**A	0.801	-	T	*NFE2L2*
*DICER1*	chr14:95568484-95568495	C**T**T**AC**TCT**GC**A	0.801	-	R, T	*MAFF, MAFK*
*DICER1*	chr14:95574241-95574252	C**TGA**TTCA**GC**A	0.879	-	R, T	*MAFK, MAFF*
*DICER1*	chr14:95599297-95599308	A**TGAC**TAAA**C**T	0.802	+	R, T	*MAFK*
*DICER1*	chr14:95606901-95606912	G**T**C**A**TTAA**GC**A	0.81	+	R, T, E	*MAFK*
*DICER1*	chr14:95606922-95606933	G**T**A**A**TTTA**GC**A	0.804	+	R, T, E	*MAFK*
*DICER1*	chr14:95606976-95606987	G**TGA**TTCAT**C**A	0.829	-	R, T, E	*MAFK*
*DROSHA*	chr5:31470983-31470994	C**TGAC**TCA**GC**A	0.941	+	T, E, WE	*MAFF, BACH1, MAFK*
*DROSHA*	chr5:31531143-31531154	A**TGAC**TCA**G**TG	0.837	-	TSS	*MAFK, MAFF*
*DROSHA*	chr5:31532503-31532514	G**TGAC**TCC**GC**G	0.841	+	TSS	*MAFK*
*DROSHA*	chr5:31537176-31537187	AA**GAC**TCA**GC**A	0.895	+	T	*MAFK*
*DROSHA*	chr5:31537251-31537262	G**TGA**GTA**G**GC	0.801	+	T	*MAFK*
*DROSHA*	chr5:31470983-31470994	C**TGAC**TCA**GC**A	0.941	+	T, E, WE	*MAFF, BACH1, MAFK*
*TARBP2*	chr12:53892564-53892575	A**TG**C**C**ACA**GC**T	0.804	+	TSS	*BACH1*
*TARBP2*	chr12:53892583-53892594	A**TG**C**C**ACA**GC**T	0.804	+	TSS, E	*BACH1*

**Table 4:** Table showing the putative ARE sequences identified in the mouse genome with a relative score over 80%. Columns and content follow the same format as Table 2.

*Gene*	Coordinates (mm10 Genome)	Motif	Relative Score	*Strand*	Regulatory Element	TFs
*Dicer1*	chr12:104699200-104699211	C**TGA**GTCA**GC**A	0.87	+	None	*Maff, Mafk*
*Dicer1*	chr12:104709040-104709051	C**TG**G**C**TCA**GC**A	0.836	+	None	*Maff, Mafk*
*Dicer1*	chr12:104709107-104709118	A**TGA**GTCAC**C**A	0.82	-	None	*Mafk*
*Dicer1*	chr12:104728875-104728886	C**TG**G**C**TCA**GC**A	0.84	+	None	*Maff, Mafk*
*Drosha*	chr15:12838880-12838891	G**TGAC**TCT**GC**A	0.94	+	None	*Nfe2l2*
*Drosha*	chr15:12848397-12848408	G**TGAC**TCA**G**GA	0.89	-	dELS	*Mafk*
*Drosha*	chr15:12858197-12858208	C**T**T**AC**TCT**GC**A	0.80	-	None	*Mafk*

## Data Availability

The data are contained within the article and supplementary materials.

## Supplementary Materials

The following supporting information can be downloaded at: https://www.mdpi.com/article/10.3390/antiox12030641/s1, Figure S1: Verification of MEF genotype and DMF treatment, Suppl. Table S1—List of transcripts scanned for putative ARE sequences in the human genome (hg19); Suppl. Table S2—List of transcripts scanned for putative ARE sequences in the mouse genome (mm10); Suppl. Table S3. List of primers used in this study; Suppl. Table S4. List of antibodies used in this study

## References

[1] Stephens K.E., Miaskowski C.A., Levine J.D., Pullinger C.R., Aouizerat B.E. (2013). Epigenetic regulation and measurement of epigenetic changes. Biol. Res. Nurs..

[2] Jaenisch R., Bird A. (2003). Epigenetic regulation of gene expression: How the genome integrates intrinsic and environmental signals. Nat. Genet..

[3] Kreuz S., Fischle W. (2016). Oxidative stress signaling to chromatin in health and disease. Epigenomics.

[4] Dinkova-Kostova A.T., Kostov R.V., Kazantsev A.G. (2018). The role of Nrf2 signaling in counteracting neurodegenerative diseases. FEBS J..

[5] Cuadrado A. (2022). Brain-Protective Mechanisms of Transcription Factor NRF2: Toward a Common Strategy for Neurodegenerative Diseases. Annu. Rev. Pharmacol. Toxicol..

[6] Bukke V.N., Moola A., Serviddio G., Vendemiale G., Bellanti F. (2022). Nuclear factor erythroid 2-related factor 2-mediated signaling and metabolic associated fatty liver disease. World J. Gastroenterol..

[7] Ngo V., Duennwald M.L. (2022). Nrf2 and Oxidative Stress: A General Overview of Mechanisms and Implications in Human Disease. Antioxidants.

[8] Itoh K., Chiba T., Takahashi S., Ishii T., Igarashi K., Katoh Y., Oyake T., Hayashi N., Satoh K., Hatayama I. (1997). An Nrf2/small Maf heterodimer mediates the induction of phase II detoxifying enzyme genes through antioxidant response elements. Biochem. Biophys. Res. Commun..

[9] Itoh K., Wakabayashi N., Katoh Y., Ishii T., Igarashi K., Engel J.D., Yamamoto M. (1999). Keap1 represses nuclear activation of antioxidant responsive elements by Nrf2 through binding to the amino-terminal Neh2 domain. Genes Dev..

[10] Baird L., Yamamoto M. (2020). The Molecular Mechanisms Regulating the KEAP1-NRF2 Pathway. Mol. Cell. Biol..

[11] McMahon M., Thomas N., Itoh K., Yamamoto M., Hayes J.D. (2004). Redox-regulated turnover of Nrf2 is determined by at least two separate protein domains, the redox-sensitive Neh2 degron and the redox-insensitive Neh6 degron. J. Biol. Chem..

[12] Yu S., Khor T.O., Cheung K.L., Li W., Wu T.Y., Huang Y., Foster B.A., Kan Y.W., Kong A.N. (2010). Nrf2 expression is regulated by epigenetic mechanisms in prostate cancer of TRAMP mice. PLoS ONE.

[13] Taheri Z., Asadzadeh Aghdaei H., Irani S., Modarressi M.H., Zahra N. (2020). Evaluation of the Epigenetic Demethylation of NRF2, a Master Transcription Factor for Antioxidant Enzymes, in Colorectal Cancer. Rep. Biochem. Mol. Biol..

[14] Shah N.M., Rushworth S.A., Murray M.Y., Bowles K.M., MacEwan D.J. (2013). Understanding the role of NRF2-regulated miRNAs in human malignancies. Oncotarget.

[15] Quiles J.M., Pepin M.E., Sunny S., Shelar S.B., Challa A.K., Dalley B., Hoidal J.R., Pogwizd S.M., Wende A.R., Rajasekaran N.S. (2021). Identification of Nrf2-responsive microRNA networks as putative mediators of myocardial reductive stress. Sci. Rep..

[16] Kaundal R.K., Datusalia A.K., Sharma S.S. (2022). Posttranscriptional regulation of Nrf2 through miRNAs and their role in Alzheimer’s disease. Pharmacol. Res..

[17] Milanesi E., Dobre M., Cucos C.A., Rojo A.I., Jiménez-Villegas J., Capetillo-Zarate E., Matute C., Piñol-Ripoll G., Manda G., Cuadrado A. (2021). Whole Blood Expression Pattern of Inflammation and Redox Genes in Mild Alzheimer’s Disease. J. Inflamm. Res..

[18] Hoffman M.M., Ernst J., Wilder S.P., Kundaje A., Harris R.S., Libbrecht M., Giardine B., Ellenbogen P.M., Bilmes J.A., Birney E. (2013). Integrative annotation of chromatin elements from ENCODE data. Nucleic Acids Res..

[19] Moore J.E., Purcaro M.J., Pratt H.E., Epstein C.B., Shoresh N., Adrian J., Kawli T., Davis C.A., Dobin A., Kaul R. (2020). Expanded encyclopaedias of DNA elements in the human and mouse genomes. Nature.

[20] Zou Z., Ohta T., Miura F., Oki S. (2022). ChIP-Atlas 2021 update: A data-mining suite for exploring epigenomic landscapes by fully integrating ChIP-seq, ATAC-seq and Bisulfite-seq data. Nucleic Acids Res..

[21] Quinlan A.R., Hall I.M. (2010). BEDTools: A flexible suite of utilities for comparing genomic features. Bioinform..

[22] Dale R.K., Pedersen B.S., Quinlan A.R. (2011). Pybedtools: A flexible Python library for manipulating genomic datasets and annotations. Bioinform. (Oxf. Engl.).

[23] Castro-Mondragon J.A., Riudavets-Puig R., Rauluseviciute I., Lemma R.B., Turchi L., Blanc-Mathieu R., Lucas J., Boddie P., Khan A., Manosalva Pérez N. (2022). JASPAR 2022: The 9th release of the open-access database of transcription factor binding profiles. Nucleic Acids Res..

[24] Martin-Hurtado A., Martin-Morales R., Robledinos-Antón N., Blanco R., Palacios-Blanco I., Lastres-Becker I., Cuadrado A., Garcia-Gonzalo F.R. (2019). NRF2-dependent gene expression promotes ciliogenesis and Hedgehog signaling. Sci. Rep..

[25] Fernández-Ginés R., Encinar J.A., Hayes J.D., Oliva B., Rodríguez-Franco M.I., Rojo A.I., Cuadrado A. (2022). An inhibitor of interaction between the transcription factor NRF2 and the E3 ubiquitin ligase adapter β-TrCP delivers anti-inflammatory responses in mouse liver. Redox Biol..

[26] Cuadrado A., Martin-Moldes Z., Ye J., Lastres-Becker I. (2014). Transcription factors NRF2 and NF-kappaB are coordinated effectors of the Rho family, GTP-binding protein RAC1 during inflammation. J. Biol. Chem..

[27] Lastres-Becker I., de Lago E., Martínez A., Fernández-Ruiz J. (2022). New Statement about NRF2 in Amyotrophic Lateral Sclerosis and Frontotemporal Dementia. Biomolecules.

[28] Li Z., Zhu W.G. (2014). Targeting histone deacetylases for cancer therapy: From molecular mechanisms to clinical implications. Int. J. Biol. Sci..

[29] Li G., Tian Y., Zhu W.G. (2020). The Roles of Histone Deacetylases and Their Inhibitors in Cancer Therapy. Front. Cell Dev. Biol..

[30] Alseksek R.K., Ramadan W.S., Saleh E., El-Awady R. (2022). The Role of HDACs in the Response of Cancer Cells to Cellular Stress and the Potential for Therapeutic Intervention. Int. J. Mol. Sci..

[31] (2018). The Role of Sirtuins in Antioxidant and Redox Signaling. Antioxid. Redox Signal..

[32] Moore L.D., Le T., Fan G. (2013). DNA Methylation and Its Basic Function. Neuropsychopharmacology.

[33] Campos A.C., Molognoni F., Melo F.H., Galdieri L.C., Carneiro C.R., D’Almeida V., Correa M., Jasiulionis M.G. (2007). Oxidative stress modulates DNA methylation during melanocyte anchorage blockade associated with malignant transformation. Neoplasia.

[34] Hedman Å.K., Zilmer M., Sundström J., Lind L., Ingelsson E. (2016). DNA methylation patterns associated with oxidative stress in an ageing population. BMC Med. Genom..

[35] Mathelier A., Zhao X., Zhang A.W., Parcy F., Worsley-Hunt R., Arenillas D.J., Buchman S., Chen C.-y., Chou A., Ienasescu H. (2013). JASPAR 2014: An extensively expanded and updated open-access database of transcription factor binding profiles. Nucleic Acids Res..

[36] Andersen M.C., Engström P.G., Lithwick S., Arenillas D., Eriksson P., Lenhard B., Wasserman W.W., Odeberg J. (2008). In silico detection of sequence variations modifying transcriptional regulation. PLoS Comput. Biol..

[37] Kwon A.T., Arenillas D.J., Worsley Hunt R., Wasserman W.W. (2012). oPOSSUM-3: Advanced analysis of regulatory motif over-representation across genes or ChIP-Seq datasets. G3 (Bethesda Md.).

[38] Hirotsu Y., Katsuoka F., Funayama R., Nagashima T., Nishida Y., Nakayama K., Engel J.D., Yamamoto M. (2012). Nrf2-MafG heterodimers contribute globally to antioxidant and metabolic networks. Nucleic Acids Res..

[39] Cuadrado A., Kugler S., Lastres-Becker I. (2018). Pharmacological targeting of GSK-3 and NRF2 provides neuroprotection in a preclinical model of tauopathy. Redox Biol..

[40] Carbonell T., Gomes A.V. (2020). MicroRNAs in the regulation of cellular redox status and its implications in myocardial ischemia-reperfusion injury. Redox Biol..

[41] Mayya V.K., Duchaine T.F. (2019). Ciphers and Executioners: How 3’-Untranslated Regions Determine the Fate of Messenger RNAs. Front. Genet..

[42] Ghini F., Rubolino C., Climent M., Simeone I., Marzi M.J., Nicassio F. (2018). Endogenous transcripts control miRNA levels and activity in mammalian cells by target-directed miRNA degradation. Nat. Commun..

[43] Thomson D.W., Dinger M.E. (2016). Endogenous microRNA sponges: Evidence and controversy. Nat. Rev. Genet..

[44] Rüegger S., Großhans H. (2012). MicroRNA turnover: When, how, and why. Trends Biochem. Sci..

[45] de la Mata M., Gaidatzis D., Vitanescu M., Stadler M.B., Wentzel C., Scheiffele P., Filipowicz W., Großhans H. (2015). Potent degradation of neuronal miRNAs induced by highly complementary targets. EMBO Rep..

[46] Song M.Y., Lee D.Y., Chun K.S., Kim E.H. (2021). The Role of NRF2/KEAP1 Signaling Pathway in Cancer Metabolism. Int. J. Mol. Sci..

[47] Zhou J.W., Wang M., Sun N.X., Qing Y., Yin T.F., Li C., Wu D. (2019). Sulforaphane-induced epigenetic regulation of Nrf2 expression by DNA methyltransferase in human Caco-2 cells. Oncol. Lett..

[48] Sciacovelli M., Gonçalves E., Johnson T.I., Zecchini V.R., da Costa A.S., Gaude E., Drubbel A.V., Theobald S.J., Abbo S.R., Tran M.G. (2016). Fumarate is an epigenetic modifier that elicits epithelial-to-mesenchymal transition. Nature.

[49] Maltby V.E., Lea R.A., Ribbons K.A., Sanders K.A., Kennedy D., Min M., Scott R.J., Lechner-Scott J. (2018). DNA methylation changes in CD4(+) T cells isolated from multiple sclerosis patients on dimethyl fumarate. Mult. Scler. J.-Exp. Transl. Clin..

[50] Carlström K.E., Ewing E., Granqvist M., Gyllenberg A., Aeinehband S., Enoksson S.L., Checa A., Badam T.V.S., Huang J., Gomez-Cabrero D. (2019). Therapeutic efficacy of dimethyl fumarate in relapsing-remitting multiple sclerosis associates with ROS pathway in monocytes. Nat. Commun..

[51] Pouremamali F., Pouremamali A., Dadashpour M., Soozangar N., Jeddi F. (2022). An update of Nrf2 activators and inhibitors in cancer prevention/promotion. Cell Commun. Signal..

[52] Castro-Muñoz L.J., Ulloa E.V., Sahlgren C., Lizano M., De La Cruz-Hernández E., Contreras-Paredes A. (2023). Modulating epigenetic modifications for cancer therapy (Review). Oncol. Rep..

[53] Wang N., Ma T., Yu B. (2023). Targeting epigenetic regulators to overcome drug resistance in cancers. Signal Transduct. Target. Ther..

[54] Potaczek D.P., Alashkar Alhamwe B., Miethe S., Garn H. (2022). Epigenetic Mechanisms in Allergy Development and Prevention. Handb. Exp. Pharmacol..

[55] Sundaramoorthy T.H., Castanho I. (2022). The Neuroepigenetic Landscape of Vertebrate and Invertebrate Models of Neurodegenerative Diseases. Epigenetics Insights.

[56] Yildiz C.B., Zimmer-Bensch G. (2022). Role of DNMTs in the Brain. Adv. Exp. Med. Biol..

[57] Zhang S., Duan S., Xie Z., Bao W., Xu B., Yang W., Zhou L. (2022). Epigenetic Therapeutics Targeting NRF2/KEAP1 Signaling in Cancer Oxidative Stress. Front. Pharmacol..

[58] Camiña N., Penning T.M. (2022). Genetic and epigenetic regulation of the NRF2-KEAP1 pathway in human lung cancer. Br. J. Cancer.

[59] Bovilla V.R., Kuruburu M.G., Bettada V.G., Krishnamurthy J., Sukocheva O.A., Thimmulappa R.K., Shivananju N.S., Balakrishna J.P., Madhunapantula S.V. (2021). Targeted Inhibition of Anti-Inflammatory Regulator Nrf2 Results in Breast Cancer Retardation In Vitro and In Vivo. Biomedicines.

[60] Ballout F., Lu H., Chen Z., Hu T., Chen L., Washington M.K., El-Rifai W., Peng D. (2022). Targeting NRF2 Sensitizes Esophageal Adenocarcinoma Cells to Cisplatin through Induction of Ferroptosis and Apoptosis. Antioxidants.

[61] El-Naggar A.M., Somasekharan S.P., Wang Y., Cheng H., Negri G.L., Pan M., Wang X.Q., Delaidelli A., Rafn B., Cran J. (2019). Class I HDAC inhibitors enhance YB-1 acetylation and oxidative stress to block sarcoma metastasis. EMBO Rep..

[62] McMahon M., Campbell K.H., MacLeod A.K., McLaughlin L.A., Henderson C.J., Wolf C.R. (2014). HDAC inhibitors increase NRF2-signaling in tumour cells and blunt the efficacy of co-adminstered cytotoxic agents. PLoS ONE.

[63] Xue F., Huang J.W., Ding P.Y., Zang H.G., Kou Z.J., Li T., Fan J., Peng Z.W., Yan W.J. (2016). Nrf2/antioxidant defense pathway is involved in the neuroprotective effects of Sirt1 against focal cerebral ischemia in rats after hyperbaric oxygen preconditioning. Behav. Brain Res..

[64] Zhang J., Pan W., Zhang Y., Tan M., Yin Y., Li Y., Zhang L., Han L., Bai J., Jiang T. (2022). Comprehensive overview of Nrf2-related epigenetic regulations involved in ischemia-reperfusion injury. Theranostics.

[65] Hockly E., Richon V.M., Woodman B., Smith D.L., Zhou X., Rosa E., Sathasivam K., Ghazi-Noori S., Mahal A., Lowden P.A. (2003). Suberoylanilide hydroxamic acid, a histone deacetylase inhibitor, ameliorates motor deficits in a mouse model of Huntington’s disease. Proc. Natl. Acad. Sci. USA.

[66] Petri S., Kiaei M., Kipiani K., Chen J., Calingasan N.Y., Crow J.P., Beal M.F. (2006). Additive neuroprotective effects of a histone deacetylase inhibitor and a catalytic antioxidant in a transgenic mouse model of amyotrophic lateral sclerosis. Neurobiol. Dis..

[67] Li L.-H., Peng W.-N., Deng Y., Li J.-J., Tian X.-R. (2020). Action of trichostatin A on Alzheimer’s disease-like pathological changes in SH-SY5Y neuroblastoma cells. Neural Regen. Res..

[68] Lastres-Becker I., Innamorato N.G., Jaworski T., Rabano A., Kugler S., Van Leuven F., Cuadrado A. (2014). Fractalkine activates NRF2/NFE2L2 and heme oxygenase 1 to restrain tauopathy-induced microgliosis. Brain.

[69] Lastres-Becker I., Ulusoy A., Innamorato N.G., Sahin G., Rábano A., Kirik D., Cuadrado A. (2012). α-Synuclein expression and Nrf2 deficiency cooperate to aggravate protein aggregation, neuronal death and inflammation in early-stage Parkinson’s disease. Hum. Mol. Genet..

[70] Cao H., Wang L., Chen B., Zheng P., He Y., Ding Y., Deng Y., Lu X., Guo X., Zhang Y. (2015). DNA Demethylation Upregulated Nrf2 Expression in Alzheimer’s Disease Cellular Model. Front. Aging Neurosci..

[71] Gao Q., Chen F., Zhang L., Wei A., Wang Y., Wu Z., Cao W. (2022). Inhibition of DNA methyltransferase aberrations reinstates antioxidant aging suppressors and ameliorates renal aging. Aging Cell.

[72] Zhou Z., Li H.Q., Liu F. (2018). DNA Methyltransferase Inhibitors and their Therapeutic Potential. Curr. Top. Med. Chem..

[73] Kurinna S., Werner S. (2015). NRF2 and microRNAs: New but awaited relations. Biochem. Soc. Trans..

[74] Xu Y., Huang X., Luo Q., Zhang X. (2021). MicroRNAs Involved in Oxidative Stress Processes Regulating Physiological and Pathological Responses. MicroRNA.

[75] Cheng X., Ku C.H., Siow R.C. (2013). Regulation of the Nrf2 antioxidant pathway by microRNAs: New players in micromanaging redox homeostasis. Free Radic. Biol. Med..

[76] Lettieri-Barbato D., Aquilano K., Punziano C., Minopoli G., Faraonio R. (2022). MicroRNAs, Long Non-Coding RNAs, and Circular RNAs in the Redox Control of Cell Senescence. Antioxidants.

[77] Bu H., Wedel S., Cavinato M., Jansen-Dürr P. (2017). MicroRNA Regulation of Oxidative Stress-Induced Cellular Senescence. Oxidative Med. Cell. Longev..

[78] Espinosa-Diez C., Miguel V., Mennerich D., Kietzmann T., Sánchez-Pérez P., Cadenas S., Lamas S. (2015). Antioxidant responses and cellular adjustments to oxidative stress. Redox Biol..

[79] Xu W., Li F., Liu Z., Xu Z., Sun B., Cao J., Liu Y. (2017). MicroRNA-27b inhibition promotes Nrf2/ARE pathway activation and alleviates intracerebral hemorrhage-induced brain injury. Oncotarget.

[80] Zhao X.R., Zhang Z., Gao M., Li L., Sun P.Y., Xu L.N., Qi Y., Yin L.H., Peng J.Y. (2020). MicroRNA-27a-3p aggravates renal ischemia/reperfusion injury by promoting oxidative stress via targeting growth factor receptor-bound protein 2. Pharmacol. Res..

[81] Zhao Y., Dong D., Reece E.A., Wang A.R., Yang P. (2018). Oxidative stress-induced miR-27a targets the redox gene nuclear factor erythroid 2-related factor 2 in diabetic embryopathy. Am. J. Obstet. Gynecol..

[82] Yang H., Li T.W., Zhou Y., Peng H., Liu T., Zandi E., Martínez-Chantar M.L., Mato J.M., Lu S.C. (2015). Activation of a novel c-Myc-miR27-prohibitin 1 circuitry in cholestatic liver injury inhibits glutathione synthesis in mice. Antioxid. Redox Signal..

[83] Woodbury M.E., Freilich R.W., Cheng C.J., Asai H., Ikezu S., Boucher J.D., Slack F., Ikezu T. (2015). miR-155 Is Essential for Inflammation-Induced Hippocampal Neurogenic Dysfunction. J. Neurosci. Off. J. Soc. Neurosci..

[84] Jian Y., Song Z., Ding Z., Wang J., Wang R., Hou X. (2022). Upregulation of Spinal miR-155-5p Contributes to Mechanical Hyperalgesia by Promoting Inflammatory Activation of Microglia in Bone Cancer Pain Rats. Life.

[85] Gaudet A.D., Mandrekar-Colucci S., Hall J.C., Sweet D.R., Schmitt P.J., Xu X., Guan Z., Mo X., Guerau-de-Arellano M., Popovich P.G. (2016). miR-155 Deletion in Mice Overcomes Neuron-Intrinsic and Neuron-Extrinsic Barriers to Spinal Cord Repair. J. Neurosci. Off. J. Soc. Neurosci..

