# Supplementary material for: The Transcription Factor NRF2 Has Epigenetic Regulatory Functions Modulating HDACs, DNMTs, and miRNA Biogenesis
Source: Antioxidants (Basel). 2023 Mar 4;12(3):641. doi: 10.3390/antiox12030641 (PMC10045347; doi:10.3390/antiox12030641)
Supplement: Supplementary file 1 [file antioxidants-12-00641-s001.zip › antioxidants-2251102-supplementary.pdf]

Suppl. Figure 1

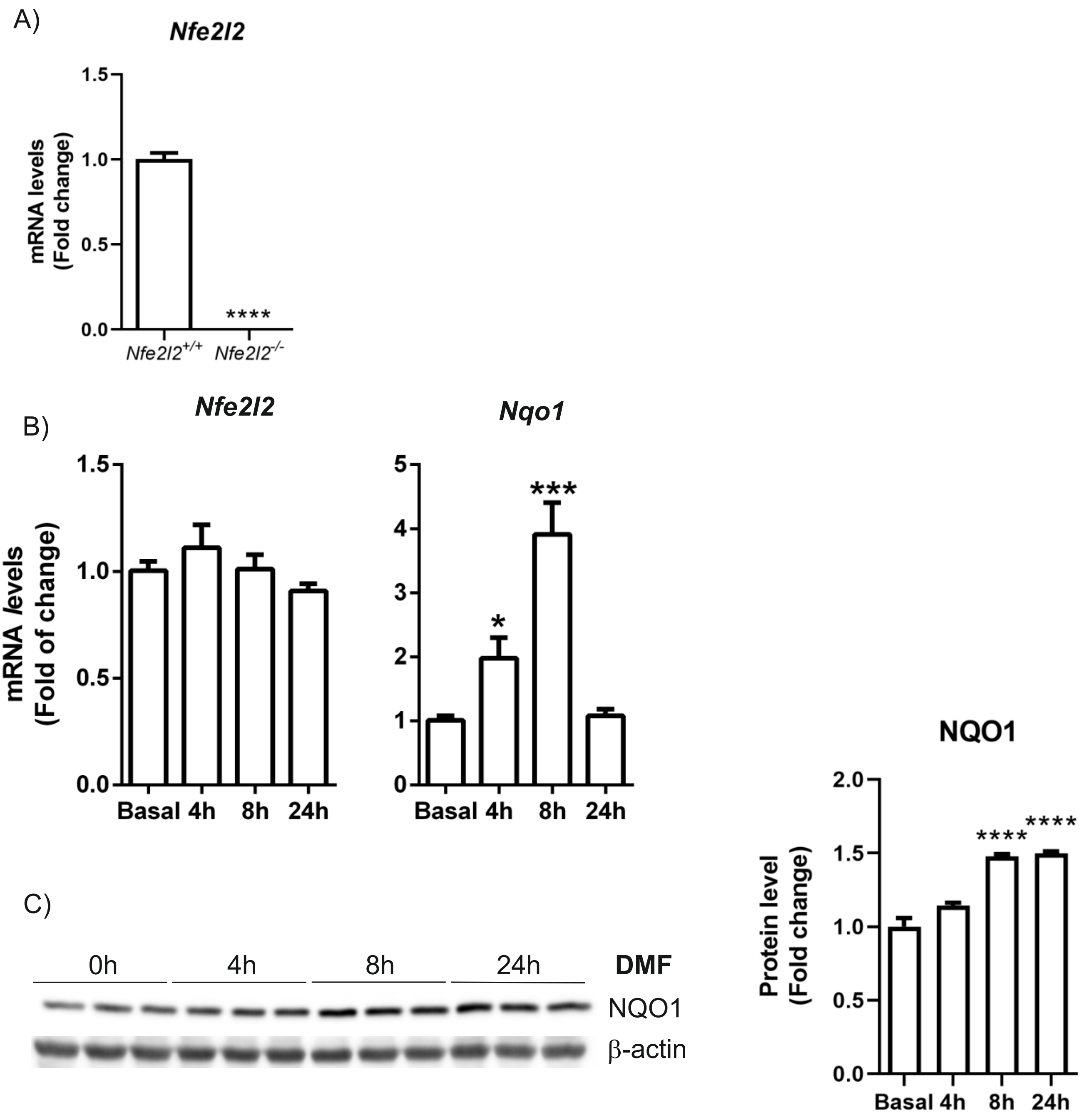

Suppl. Table 1 – List of transcripts scanned for putative ARE sequences in the human genome (hg19). HUGO Gene Nomenclature Committee symbols and NCBI Refseq mRNA ID accession numbers are indicated for each gene and transcript.

	**Human genome (hg19)**
HGNC Symbol	**Refseq mRNA ID accesion number**
DGCR8	NM_001190326, NM_022720
DICER 1	NM_001195573, NM_001271282, NM_001291628, NM_001395677, NM_001395678, NM_001395679, NM_001395680, NM_001395682, NM_001395683, NM_001395684, NM_001395685, NM_001395686, NM_001395687, NM_001395688, NM_001395689, NM_001395690, NM_001395691, NM_001395692, NM_001395693, NM_001395694, NM_001395695, NM_001395696, NM_001395697, NM_001395698, NM_001395700, NM_030621, NM_177438
DNMT1	NM_001130823, NM_001318730, NM_001318731, NM_001379
DNMT3A	NM_001320892, NM_001320893, NM_001375819, NM_022552, NM_153759, NM_175629, NM_175630
DNMT3B	NM_001207055, NM_001207056, NM_006892, NM_175848, NM_175849, NM_175850
DROSHA	NM_001100412, NM_001382508, NM_013235
HDAC1	NM_004964
HDAC2	NM_001527
HDAC3	NM_001355039, NM_001355040, NM_001355041, NM_003883
SIRT1	NM_001142498, NM_001314049, NM_012238
TARBP2	NM_004178, NM_134323, NM_134324

Suppl. Table 2 – List of transcripts scanned for putative ARE sequences in the mouse genome (mm10). Mouse Genome Informatics symbols and NCBI Refseq mRNA ID accession numbers are indicated for each gene and transcript.

	**Mouse genome (mm10)**
MGI Symbol	**Refseq mRNA ID accesion number**
Dgcr8	NM_033324
Dicer1	NM_148948
Dnmt1	NM_001404685, NM_010066, NM_001199431, NM_001314011, NM_001199432, NM_001199433
Dnmt3a	NM_007872, NM_001271753, NM_153743
Dnmt3b	NM_001271744, NM_001271745, NM_001003961, NM_001003960, NM_001271747, NM_001003963, NM_001271746, NM_010068, NM_001122997
Drosha	NM_001130149, NM_026799
Hdac1	NM_008228
Hdac2	NM_008229
Hdac3	NM_010411
Sirt1	NM_001159589, NM_019812
Tarbp2	NM_001253795, NM_009319

Suppl. Table 3. List of primers used in this study.

Gene Product	Forward Primer	Reverse Primer
Actb	5′ TCCTTCCTGGGCATGGAG 3′	5′ AGGAGGAGCAATGATCTTGATCTT 3′
Dicer1	5′ TGAGAAGCAAAAAGGTCAGCA 3′	5′ GCCAGCAAGCAGTCTTTTGT 3′
Dgcr8	5′ CAAGTGAGCCTTTTGGTGCC 3′	5′ GATGTGGTTAAAATACTCCAGTTCT 3′
Dnmt1	5′ AAGGACACGAGACATACTGC 3′	5′ TGGGTTTCCGTTTAGTGGGG 3′
Dnmt3a	5′ GACGCCAAAGAAGTGTCTGC 3′	5′ CTTGGCTATTCTGCCGTGCT 3′
Dnmt3b	5′ TCCCCCATCCATAGTGCCTT 3′	5′ TAATGCACTCCTCATACCCGC 3′
Drosha	5′ CTCTGTAGAGACTGTGAATCCTGC 3′	5′ GCTACATCTTCCGCTCACGA 3′
Gapdh	5′ CGACTTCAACAGCAACTCCCACTCTTCC 3′	5′ TGGGTGGTCCAGGGTTTCTTACTCCTT 3′
Hdac1	5′ TTCCTGCGTTCTATTCGCCC 3′	5′ AAGCCATCAAACACCGGACA 3′
Hdac2	5′ TACAGTCAAGGAGGCGGCAA 3′	5′ GGGATGACCCTGGCCATAATAA 3′
Hdac3	5′ TCAGCCCCACCAATATGCAG 3′	5′ TGTAACGGGAGCAGAACTCG 3′
Nqo1	5′ GGTAGCGGCTCCATGTACTC 3′	5′ CATCCTTCCAGGATCTGCAT 3′
Nfe2l2	5′ CCCGAAGCACCCTGAAGGCA 3′	5′ CCAGGCGGTGGGTCTCCGTA 3′
Sirt1	5′ GACAGAACGTCACACGCCAG 3′	5′ AGCTCAGGTGGAGGAATTGT 3′
Tarbp2	5′ GACCTTCTCAAAGCCGAGGG 3′	5′ GAAAAAGAACTGCTGTCCTCCA 3′

Suppl. Table 4. List of antibodies used in this study.

Antibody	Source	Catalog Number	Dilution
β-ACTIN (C4)	Santa Cruz Biotechnologies	sc-47778	1:1000
β-TUBULIN (TUB2.1)	SIGMA	T4026	1:3000
DGCR8	proteintech	60084-1-Ig	1:1000
DICER1 (F-10)	Santa Cruz Biotechnologies	sc-136979	1:1000
DNMT1	EPIGENTEK	60B122.1	1:1000
DNMT3a (H-295)	Santa Cruz Biotechnologies	sc-20703	1:500
DNMT3b (G-9)	Santa Cruz Biotechnologies	sc-376043	1:500
GAPDH (6C5)	Santa Cruz Biotechnologies	sc-32233	1:1000
HDAC1 (H-51)	Santa Cruz Biotechnologies	sc-7872	1:500
HDAC2 (C-8)	Santa Cruz Biotechnologies	sc-9959	1:500
NQO1	Abcam	ab2346	1:1000
DROSHA (C-7)	Santa Cruz Biotechnologies	sc-393591	1:500
SIRT1	Abcam	ab12193	1.2000
TARBP2	SIGMA	SAB1406507	1:500
VINCULIN (N3C1)	Gentex	GTX109749	1:1000
